# Laboratory evaluation of molecular xenomonitoring using mosquito and tsetse fly excreta/feces to amplify *Plasmodium*, *Brugia*, and *Trypanosoma* DNA

**DOI:** 10.12688/gatesopenres.13093.2

**Published:** 2020-06-03

**Authors:** Nils Pilotte, Darren A.N. Cook, Joseph Pryce, Michael F. Zulch, Corrado Minetti, Lisa J. Reimer, Steven A. Williams

**Affiliations:** 1 Department of Biological Sciences, Smith College, Northampton, Massachusetts, 01063, USA; 2 Molecular and Cellular Biology Program, University of Massachusetts, Amherst, Massachusetts, 01003, USA; 3 Department of Vector Biology, Liverpool School of Tropical Medicine, Liverpool, L3 5QA, UK

**Keywords:** molecular xenomonitoring, excreta/feces, lymphatic filariasis, malaria, human African trypanosomiasis, mosquito, surveillance

## Abstract

**Background:  **Results from an increasing number of studies suggest that mosquito excreta/feces (E/F) testing has considerable potential to serve as a supplement for traditional molecular xenomonitoring techniques. However, as the catalogue of possible use-cases for this methodology expands, and the list of amenable pathogens grows, a number of fundamental methods-based questions remain. Answering these questions is critical to maximizing the utility of this approach and to facilitating its successful implementation as an effective tool for molecular xenomonitoring.

**Methods:  **Utilizing E/F produced by mosquitoes or tsetse flies experimentally exposed to *Brugia malayi*, *Plasmodium falciparum*, or *Trypanosoma brucei brucei*, factors such as limits of detection, throughput of testing, adaptability to use with competent and incompetent vector species, and effects of additional blood feedings post parasite-exposure were evaluated.  Two platforms for the detection of pathogen signal (quantitative real-time PCR and digital PCR (dPCR)) were also compared, with strengths and weaknesses examined for each.

**Results:  **Experimental results indicated that high throughput testing is possible when evaluating mosquito E/F for the presence of either *B. malayi *or *P. falciparum* from both competent and incompetent vector mosquito species.  Furthermore, following exposure to pathogen, providing mosquitoes with a second, uninfected bloodmeal did not expand the temporal window for E/F collection during which pathogen detection was possible.  However, this collection window did appear longer in E/F collected from tsetse flies following exposure to *T. b. brucei*.  Testing also suggested that dPCR may facilitate detection through its increased sensitivity.  Unfortunately, logistical obstacles will likely make the large-scale use of dPCR impractical for this purpose.

**Conclusions:  **By examining many E/F testing variables, expansion of this technology to a field-ready platform has become increasingly feasible.  However, translation of this methodology from the lab to the field will first require field-based pilot studies aimed at assessing the efficacy of E/F screening.

## Introduction

Due largely to renewed commitments and coordinated efforts between local leaders, government officials, non-government organizations, international donors, and pharmaceutical companies, tropical disease control, elimination, and eradication efforts are making unprecedented gains ^1– 5^. Combined approaches, integrating chemotherapies, vector control strategies, education and outreach, and improvements to infrastructure are all contributing to significant programmatic successes. These successes are generating lofty goals for future interventions and expanding belief in the possibility of elimination of some tropical vector-borne diseases ^3, 6– 10^. However, as successes mount, new challenges arise, including an increasingly pressing need for capable surveillance tools. Following suspected transmission interruption, a failure of surveillance to identify and quickly react to possible incidences of disease recrudescence has significant potential to result in the forfeiture of hard-fought gains. For years, pharmaceutical partners and non-government organizations have supported programmatic efforts with substantial financial commitments, making such gains possible ^11^. However, insufficient oversight or inadequate follow-through may result in substantial disease rebound. Should such recrudescence occur in locations where transmission interruption or elimination efforts were previously believed to have succeeded, the remobilization of significant economic resources may not occur. Given these stakes, the need for low cost, non-invasive, high throughput surveillance methods is paramount to the realization of long-term programmatic goals.

Despite facing many challenges, the Global Programme for the Elimination of Lymphatic Filariasis (GPELF) continues to make remarkable progress in its efforts to meet its ambitious targets. Through the incorporation of novel strategies, such as triple drug (Ivermectin, Diethylcarbamazine, and Albendazole (IDA)) therapy ^12– 17^, global intervention efforts are beginning to realize more rapid successes. These accelerated accomplishments are allowing a growing number of countries to aspire towards World Health Organization (WHO)-sanctioned certification of lymphatic filariasis (LF) elimination. Currently, transmission assessment surveys (TAS) are used as the primary tool for measuring the successes of programmatic interventions ^18, 19^. However, pilot studies are demonstrating that TAS surveys may not be well-suited to surveillance and monitoring in IDA settings, and the need to re-examine monitoring and evaluation strategies under triple-drug interventions has been recognized ^20^. This has prompted the organization of operational research efforts aimed at developing an appropriate monitoring and evaluation strategy for triple drug stopping decisions. (Please see www.ntdsupport.org/cor-ntd/ntd-connector/term/lymphatic-filariasis for examples.) Such efforts may benefit from novel and innovative diagnostic screening methods. Of further concern, recent modeling efforts of helminth infection suggest that even in conventional treatment settings, the potential for recrudescence of infection, particularly when systemic non-compliance with mass drug administration (MDA) is significant, is likely greater than previously believed ^21^. Applying the findings of these predictive models to filarial infection, the threat of rebound likely extends for a period of many years past the WHO-suggested timeline for the completion of post-intervention TAS surveys. Warning signs of infection rebound, resulting from pockets of sustained focal transmission, are also being identified with increased frequency as “successful” LF elimination programs become further removed from the cessation of MDA ^22– 26^. These discrepancies between defined programmatic timelines and the modeled potential/empirical evidence of recrudescence suggest there exists a post-TAS “black box” period, during which infection rebound is possible but appreciable monitoring efforts have ceased. Accordingly, integrated, non-invasive, low cost, high throughput approaches to surveillance, capable of providing a “first alert” warning during such periods are critically lacking ^27, 28^.

Similar to the needs of the LF community, requirements for improved malarial surveillance are growing. Largely due to the expansion of coordinated interventions under the WHO’s Global Malaria Programme, examples of successful elimination are becoming more commonplace ^29– 31^, and many additional elimination efforts have been established or revitalized ^32– 34^. While encouraging, such successes also breed new challenges and raise new concerns. Recognizing the dangers associated with bestowing a “malaria-free status” upon a population, the WHO has cautioned against reallocating surveillance funding following programmatic achievement, advising of the need to retain adequate surveillance systems to detect recrudescence and facilitate a rapid response in the event that such rebound occurs ^35^. These statements warn of the potential for complacency that naturally follows success, resulting in the prioritization of more immediate resource needs and potentially erasing years of progress due to insufficient post-interruption monitoring activities ^35^.

Insufficient surveillance also has the potential to threaten the developing momentum of human African trypanosomiasis (HAT) elimination efforts. With 2016, 2017, and 2018 each marking record lows in reported global cases of HAT ^36– 38^, belief in the elimination of this disease as a public health concern is increasing. While gains realized through intervention have been significant and encouraging, monitoring efforts have relied heavily upon human sampling, an approach that is commonly met with increased resistance as infection prevalence declines ^39^. Further complicating matters, the causative agents of HAT, *Trypanosoma brucei* spp., are vectored by the tsetse fly. These flies are notoriously difficult to trap, and vector control strategies continue to reduce their numbers ^40– 42^. While interventions aimed at decreasing fly numbers are an increasingly important component of transmission reduction efforts ^41, 43^, declining vector populations make supplemental xenosurveillance strategies increasingly impractical. Accordingly, as aspirations for elimination grow, the importance of alternative approaches to surveillance will continue to increase.

The molecular testing of mosquito excreta/feces (E/F) for the presence of pathogens provides one approach that is a potential solution to the growing surveillance challenges plaguing GPELF, as well as global malaria and HAT elimination efforts. Previously, we described the capacity for mosquito E/F testing to vastly improve the throughput of surveillance for filarial parasites ^44^. Similarly, we demonstrated the capacity of this novel molecular xenomonitoring (MX) approach to facilitate the detection of the human malaria-causing parasites *Plasmodium vivax* and *Plasmodium falciparum* ^44, 45^, and demonstrated proof-of-concept for the “cross-vector” detection of *Trypanosoma brucei brucei* in non-vector mosquitoes ^45^. However, the expanded utility of this method will require the fine tuning of sampling strategies, centering upon the identification of appropriate target mosquito populations. We have therefore performed a series of proof-of-concept experiments aimed at further evaluating the practicality of E/F testing in preparation for field trials. Exposing laboratory-reared mosquitoes and tsetse flies to various pathogens, we have endeavored to more fully understand the variables impacting parasite signal detection within E/F collected following parasite exposure.

## Methods

### Insect rearing and blood feeding

***Mosquitoes***. Both *Anopheles gambiae* (strain G3) and *Aedes aegypti* (strain LVP) mosquitoes were internally-sourced from laboratory colonies maintained at the Liverpool School of Tropical Medicine. Mosquitoes were reared from eggs to adults and housed in BugDorm-1 insect rearing cages (Megaview Science, Taiwan; Catalogue #DP1000) at 26–27 °C with 70–80% relative humidity. Experimental exposures were performed as previously described ^45^. Briefly, adult female mosquitoes, aged 3–7 days, were sugar-starved for 18 hours prior to blood exposure in order to facilitate blood feeding. For experiments involving exposures to *Brugia malayi* or *P. falciparum*, mosquitoes were provided with either a standard human bloodmeal (obtained from the local blood bank), or a human bloodmeal spiked with a known concentration of parasites. Exposures to *B. malayi* were conducted using a Hemotek feeding system (Hemotek Ltd, Blackburn, UK; Catalogue #SP6W1-3), while *P. falciparum* exposures were performed using a glass feeder (Chemglass Life Sciences, Vineland, NJ; Catalogue #CG-1836). For experiments involving mosquito exposures to *T. b. brucei*, mosquitoes were provided with a Hemotek feeding system-supplied bloodmeal of defibrinated horse blood (TCS Biosciences, Buckingham, UK; Catalogue #HB030), with or without parasites.

***Tsetse flies***. Glossina morsitans were reared from larvae and housed in internally-made cages, constructed of lengths of plastic piping covered at each end with netting, at 27 °C ± 2 °C with a relative humidity of 65–75%. Adult flies were fed on defibrinated horse blood, with or without parasites. Feedings occurred by placing blood on an aluminum tray heated to 37 °C. Fly cages were then placed on a silicon membrane positioned directly above the blood, allowing flies to feed through the membrane.

### Parasites

***B. malayi***. Microfilaria (mf) were generously provided by the anit- *Wolbachia* Consortium, generated as part of their maintenance of the *B. malayi* lifecycle ^46^. Harvested parasites were added to human blood at the appropriate concentrations to generate experimentally desired parasite densities as described below for individual applications.

***P. falciparum***. Red blood cells containing trophozoites (3D7 strain) were combined with uninfected human serum to produce experimentally desired parasite concentrations as described below for individual applications.

***T. b. brucei***. The bloodstream form of *T. b. brucei*, strain AnTat 1.1 90:13 ^47^, was used for all experimental feedings. Parasites were cultured in HMI-11 medium supplemented with 10% fetal bovine serum at 37 °C and 5% CO _2_. Parasite densities were determined microscopically using a hemocytometer.

### Collection of excreta/feces

***B. malayi experiments***. All experiments involving *B. malayi* were performed in accordance with the previously described superhydrophobic cone collection method ^45^. Briefly, sheets of A4 printer paper were used to create cone-shaped funnels, which were coated in NeverWet (Rust-Oleum, Durham, UK). Cones were then placed inside of mesh-covered un-waxed paper beverage cups, with mosquitoes housed above the cones, allowing E/F produced by the mosquitoes to travel down the walls of the cones and pool at the base of each funnel. For these collections, GenSaver DNA Cards (GenTegra, Pleasanton, CA; Catalogue #GSD4-100) were used in place of the 1.7 mL microcentrifuge tubes that were employed when this method was previously described ^45^. For all collections, GenSaver DNA Cards, designed with four circular collection areas, were cut into quarters such that each E/F collection even occurred onto a single collection circle.

***P. falciparum experiments***. When performing experiments involving *P. falciparum*, E/F was again collected in accordance with the previously described superhydrophobic cone collection method ^45^ briefly described above. For all experiments involving *P. falciparum*, E/F samples were collected into 1.7 mL microcentrifuge tubes as previously described ^45^.

***T. b. brucei experiments***. For all experiments involving *T. b. brucei*, flies/mosquitoes were housed in 50 mL conical tubes allowing for direct deposition of E/F onto the walls of the holding vessel. During the experimental housing of vectors, tubes were covered with mesh netting, and flies/mosquitoes were transferred to new vessels at experimentally specified time intervals. While in tubes, tsetse flies were removed from tubes for feeding on uninfected defibrinated horse blood every second day as described above.

### Extraction of DNA from excreta/feces

***Following superhydrophobic cone collections onto GenSaver DNA Cards***. All samples were excised from GenSaver DNA Cards using a standard paper punch (0.64 cm round). For each sample, three punches were placed into a 2.0 mL microcentrifuge tube and the sample was recovered using the GenSolve DNA Recovery Kit (GenTegra; Catalogue #GVR-113) in accordance with the manufacturer’s suggested protocol. Following recovery, each sample was added to a MinElute column (Qiagen, Germantown, MD) for sample binding. Sample washes and DNA recovery procedures occurred utilizing the manufacturer’s recommendations. After recovering the eluate, the total volume of eluate was re-loaded onto the column a second time and again spun through the matrix to maximize sample recovery.

***Following superhydrophobic cone collections into microcentrifuge tubes***. DNA was extracted from all samples utilizing the QIAamp DNA Micro Kit (Qiagen; Catalogue #56304) following a modified version of the manufacturer’s suggested protocol. Briefly, 180 μL of Buffer AL was added to each E/F sample and tubes were vortexed on a shaking platform for 1 hr. 20 μL of Proteinase K was then added, and samples were incubated at 56 °C for 1 hr with shaking at 1,400 RPM. Following incubation, 200 μL of Buffer AL (containing 5mM carrier RNA) was added to each sample, and samples were incubated at 70 °C for 10 min. Column binding and washing steps were then performed in accordance with the manufacturer’s specifications. Following washes, elution of DNA occurred in 50 μL of Buffer AE. As described above, following the elution of DNA in 50 μL of Buffer AE, eluate was re-loaded onto the column to maximize recovery.

***Following collection into 50 mL conical tubes***. E/F was eluted from tubes through the direct addition of 7.5 mL of nuclease free water. Following the addition of water, samples underwent agitation on a vortexing platform for 30 min at 56 °C to facilitate the complete resuspension of material. Tubes were then spun at 5,000 RPM for 5 min, and the supernatant was removed from each sample. Pelleted material was resuspended in the residual volume of liquid. Following recovery, each sample underwent DNA isolation in the same manner as described above for superhydrophobic cone-based collections into microcentrifuge tubes.

### Isolation of tsetse fly midguts and preparation for DNA extraction

Tsetse fly midguts were prepared for DNA extraction following the protocol previously described by Cunningham, *et al*. ^48^. Briefly, following dissection, midguts were placed in 60 μL of 100% ethanol. 70 μL of nuclease free water was then added to each sample and samples were centrifuged at 13,000 RPM for 15 sec. Following centrifugation, 100 μL of supernatant was aspirated from each sample, and samples underwent three sequential washes with 100 μL of nuclease free water to remove residual ethanol.

### Extraction of DNA from mosquitoes and tsetse flies

In preparation for DNA isolation, 20 μL of Proteinase K, 180 μL of Buffer ATL and a 4.5 mm ball bearing were added to all carcass and midgut samples. Samples were then mechanically homogenized at a setting of 30.0 1/S for 5 min using a TissueLyser II (Qiagen). All DNA extractions were then performed using the DNeasy Blood and Tissue Kit (Qiagen; Catalogue #69581) following the extraction plate procedure. All extractions were conducted in accordance with the manufacturer’s suggested protocol.

### Real-time PCR

All quantitative real-time PCR (qPCR) testing for the presence/absence of *B. malayi* occurred using the StepOnePlus Real-Time PCR System (ThermoFisher Scientific, Waltham, MA) and was performed using primers and probe previously described for use with the Bm HhaI real-time PCR assay ^49^. Cycling conditions consisted of an initial hold at 50 °C for 2 min, followed by a 95 °C incubation for 10 min. These incubations were followed by 45 cycles of sequential denaturation and annealing/extension steps at 95 °C for 15 sec, and 60 °C for 1 min respectively. All qPCR testing for the presence of *P. falciparum* also occurred using the StepOnePlus Real-Time PCR System and employed the recently described *Pf* TR1 assay in accordance with suggested reagent concentrations ^50^. Cycling conditions for *P. falciparum* detection were identical to those described above for *B. malayi* detection. All reactions for *B. malayi* and *P. falciparum* detection were performed in 25 μL total volumes with 5 μL of template. Genomic DNA positive PCR controls (200 pg/well) and no template control (NTC) wells were run on each reaction plate. Each reaction was conducted using 12.5 μL of TaqPath ProAmp Master Mix (ThermoFisher Scientific; Catalogue #A30867) and a Cq cut-off value of 45 was employed. Depending upon the experiment, samples were tested in duplicate or triplicate reactions and mean Cq values were reported as was the number of positive replicates.

All qPCR testing for the presence/absence of *T. b. brucei* was performed using the Rotor-Gene Q Instrument (Qiagen) and made use of the previously described Tb117 assay primers at concentrations of 400 nM ^48^. All reactions for the detection of *T. b. brucei* were performed in 10 μL volumes, using 5 μL of Type-it HRM PCR Master Mix (Qiagen; Catalogue #206542) and 4 μL of DNA template. Cycling conditions consisted of an initial hold at 96 °C for 5 min, followed by 35 cycles of 95 °C for 15 sec, 60 °C for 30 sec, and 72 °C for 10 sec. As this assay makes use of a saturating fluorescent dye (similar to SYBR Green assay chemistry) a dissociation step was then performed utilizing a temperature gradient gradually increasing from 55 °C to 95 °C. Genomic DNA positive PCR controls (5 genome equivalents/well) and NTC wells were run on each reaction plate. All *T. b. brucei* testing occurred in duplicate and both mean Cq values and the number of positive replicates were reported.

### Digital PCR

 All digital PCR (dPCR) reactions were performed on the QuantStudio 3D Digital PCR instrument using V2 chips (ThermoFisher Scientific; Catalogue #A26359). Reactions were conducted using the same *P. falciparum* primer-probe pairings selected for qPCR with identical working concentrations. All reactions were prepared in 15 μL volumes, with 14.5 μL of this prepared reaction mix loaded onto each chip for analysis. Individual reaction mixes contained 7.5 μL of QuantStudio 3D Digital PCR Master Mix v2 (ThermoFisher Scientific; Catalogue #A26316), the appropriate concentrations of primers and probe, and 5 μL of template. Cycling conditions consisted of two initial holds at 96 °C for 10 min and 50 °C for 30 sec. These holds were followed by 39 cycles of 60 °C for 2 min, 98 °C for 30 sec, and 60 °C for 2 min. Two replicate chips were analyzed when testing each sample. For each iteration of samples tested, two NTC chips containing nuclease-free water in place of template were analyzed alongside experimental samples. For a given iteration, NTC results were used to determine positivity by setting the fluorescence threshold for the entire sample set at 125% of the fluorescence reading generated by the NTC well producing the greatest level of background. When visualizing QuantStudio 3D output graphically, signal-producing wells containing true positives should be located in positions along the x-axis directly above the population of wells that failed to amplify. For this reason, as well as for consistency, and for the maintenance of a conservative approach to positivity determination, only wells with a fluorescence unit values of -240 to 240 along the x-axis were analyzed.

### Limits of detection for parasite signal from pooled E/F

***B. malayi***. Utilizing previously published temporal collection windows ^45^, infected blood exposures were conducted in order to evaluate the capacity to detect *B. malayi* signal in the E/F of individual competent ( *Ae. aegypti*) and incompetent ( *An. gambiae*) vectors. To evaluate limits of detection, mosquitoes were exposed to either 2,000 *B. malayi* mf/mL, or 5,000 *B. malayi* mf/mL. For each species of mosquito, either 10 or 11 replicate exposures were performed and the accumulated E/F was collected at the 48- and 72-hour time points post-exposure. An additional 5 mosquitoes were provided with naïve bloodmeals to serve as uninfected controls, and collections from naïve mosquitoes occurred at the same post-exposure time points. All collections were performed using superhydrophobic cones and E/F was collected onto GenSaver DNA Cards. Following collection, DNA was isolated from all E/F samples and the resulting extracts were analyzed using qPCR.

***P. falciparum***. As was done to evaluate limits of detection for *B. malayi*, the capacity to detect *P. falciparum* signal in the E/F of mosquitoes exposed to varying blood concentrations of parasite was examined. Exposures of individually housed *An. gambiae* mosquitoes occurred at 5,000 trophozoites/μL (0.1% parasitemia), 500 trophozoites/μL (0.01% parasitemia), and 50 trophozoites/μL (0.001% parasitemia), with between nine and 14 mosquitoes successfully undergoing exposure at each experimental concentration. An additional five mosquitoes were provided with a parasite-naïve bloodmeal for control purposes. Following exposure, all mosquitoes were individually housed in paper cups facilitating superhydrophobic cone-based collections of E/F into 1.7 mL microcentrifuge tubes. At the 48-hour time point, and again at 72 hours post-exposure, mosquitoes were transferred to new cups and all deposited E/F was prepared for qPCR analysis. In order to investigate whether dPCR could be used as a means of extending detection windows, E/F samples also underwent analysis by dPCR.

***T. b. brucei***. Previous work has demonstrated the successful detection of *T. b. brucei* from the E/F produced by pools of 10 mosquitoes following exposure to parasites ^45^. However, the capacity for detection of *T. b. brucei* signal from the E/F of individual mosquitoes has not yet been evaluated. The capacity for tsetse fly E/F to similarly allow for *T. b. brucei* signal detection has also yet to be appraised. To investigate these possibilities, *An. gambiae* and *G. morsitans* were exposed to defibrinated horse blood containing either “high dose” (10 ^5^ trypanosomes/mL) or “low dose” (10 ^3^ trypanosomes/mL) concentrations of parasites. Following exposure for 24 hours, individual flies and mosquitoes were transferred into 50 mL conical tubes for the collection of E/F. In total, E/F samples from 25 flies and 25 mosquitoes exposed to each dose of parasite were evaluated. An additional five flies and five mosquitoes provided with a bloodmeal that was naïve for parasite were included for control purposes. Following an initial 48-hour housing, flies/mosquitoes were transferred to new tubes and soiled tubes were collected for molecular analysis. This collection process was repeated at 96 hours post-exposure, again at 144 hours post-exposure, and finally at 192 hours post-exposure. DNA was then extracted from all collected samples and real-time PCR analysis was performed. Following the 192-hour time point, flies and mosquitoes were sacrificed, and both fly midguts and mosquito carcasses underwent DNA extraction and qPCR analysis.

### Demonstration of high throughput detection of *P. falciparum* signal from Pooled E/F

 Prior experimentation has revealed the improved throughput of detection for *B. malayi* using E/F ^44^. To investigate if throughput would also improve when detecting *P. falciparum*, pools of 49 *An. gambiae* mosquitoes were provided with a parasite-naïve bloodmeal and E/F from each pool was allowed to collect into a single microcentrifuge tube for 72 hours using a hydrophobic cone. Following 72 hours, this tube was transferred to the collecting position beneath a new cone, allowing for the collection of E/F from a single mosquito exposed to *P. falciparum* at a parasitemia of 0.1%. Accumulation of E/F from this single exposed mosquito continued until the 72-hour post-exposure time point, after which the tube was removed for downstream DNA extraction and qPCR analysis. All samples were tested in triplicate, and positivity was defined as the occurrence of a positive result in two or more reactions with a Cq value ≤ 40. Ten replicate pools were prepared. Additionally, E/F from 10 individual mosquitoes, also exposed to *P. falciparum* at the same 0.1% parasitemia, were collected for comparative purposes.

### Effects on parasite detection of a second blood feeding with pathogen-naïve blood

**Figure 1.**
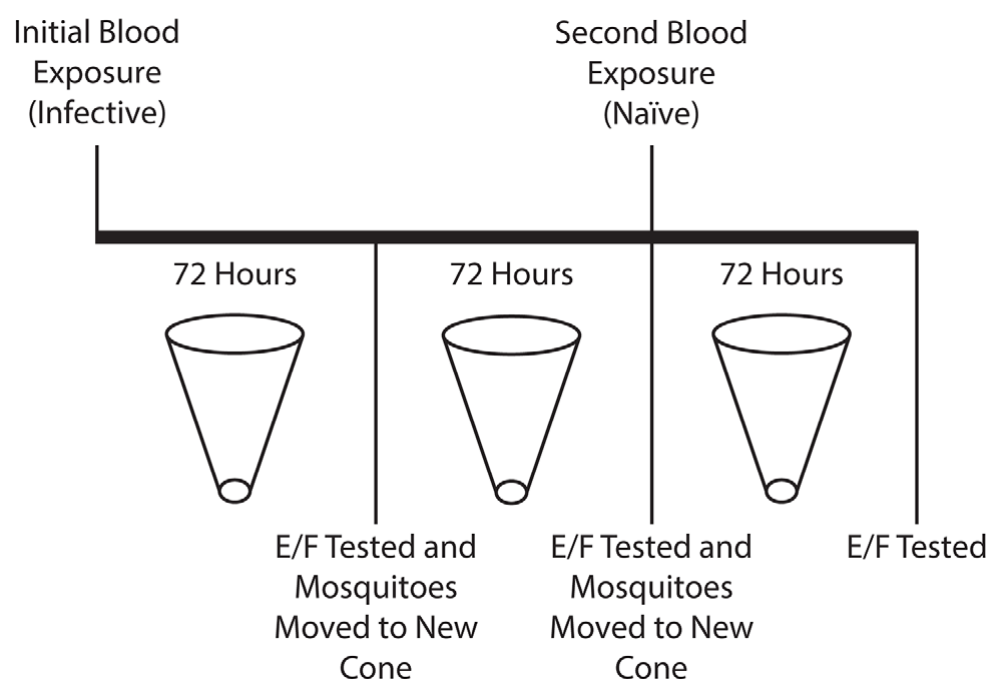
Schematic of the re-exposure experiment. Following an initial exposure to *Plasmodium falciparum-*positive blood, excreta/feces (E/F) was collected from mosquito pools for 72 hours. Following this time period, E/F samples were collected for qPCR analysis and mosquitoes were transferred to new superhydrophobic cones, where they were held for an additional 72 hours. At the conclusion of this period, E/F samples were again collected for qPCR analysis and mosquitoes were provided with a second bloodmeal, this time naïve of parasite. Mosquitoes were then transferred to a third superhydrophobic cone, where excretion continued for an additional 72 hours. Following this final incubation period, E/F was again collected for qPCR analysis.

 To evaluate whether the provision of a second bloodmeal following an initial infected blood exposure would facilitate an extended window of parasite detection, three pools of 10 *An. gambiae* mosquitoes were exposed to *P. falciparum*-containing blood at a parasitemia of 0.01%, and an additional control pool, also containing 10 *An. gambiae* mosquitoes, was provided with a parasite-naïve bloodmeal. Using a superhydrophobic cone, E/F from each pool of mosquitoes was collected into a microcentrifuge tube for a 72-hour period following exposure. Mosquito pools were then transferred to new cones, and E/F was allowed to accumulate for an additional 72 hours into a new microcentrifuge tube. At 144 hours post-feeding, mosquitoes were again transferred to new cones/tubes and a naïve bloodmeal was provided. Following this second blood exposure, an additional 72-hour collection was performed. All collected samples then underwent DNA extraction and triplicate testing by qPCR ( Figure 1).

### Statistical analysis

Statistical differences between groups were determined by means of a Student’s two-tailed *t*-test performed using GraphPad’s “ *t* test calculator” freely available from graphpad.com. A *p* value < 0.05 was considered to be statistically significant (* *p* < 0.05, ** *p* < 0.01, *** *p* < 0.001). Where appropriate, confidence intervals were calculated using the previously described E. B. Wilson method ^51, 52^ utilizing software freely available at http://vassarstats.net/prop1.html.

## Results

Raw qPCR and dPCR data underlying the below results are available as underlying data ^53^.

### Limits of detection for parasite signal from pooled E/F

**Figure 2.**
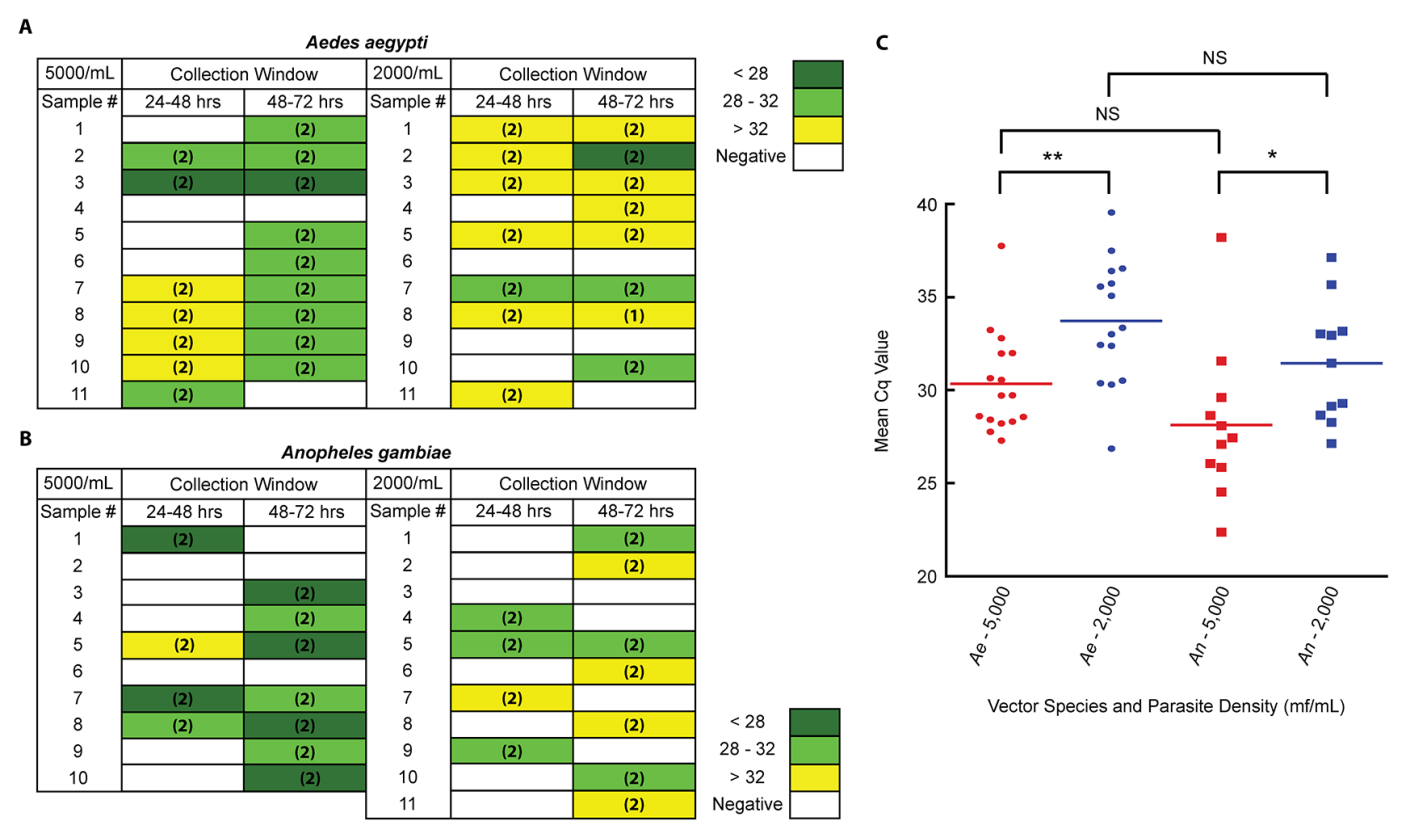
Detection windows for parasites in the excreta/feces (E/F) of mosquitoes following a *B. malayi-*containing bloodmeal. Individual ( **A**) *Aedes aegypti* (competent vector) mosquitoes and ( **B**) *Anopheles gambiae* (incompetent vector) mosquitoes were exposed to a *Brugia malayi*-containing bloodmeal at a parasitemia of either 5000 mf/mL or 2000 mf/mL. E/F was then collected from each mosquito, individually, in 24-hour time blocks. Collected E/F samples were then tested in duplicate qPCR reactions. Colors represent Cq values, and numbers in parenthesis indicate the number of positive qPCR replicates. ( **C**) A comparison of the mean Cq values from all positive E/F samples produced by both mosquito species at both parasitemias. Significance, as determined by the results of unpaired *t* tests, is provided. * *p* < 0.05, ** *p* < 0.01, NS = not significant.

***B. malayi***. Individual competent vector ( *Ae. aegypti*) and incompetent vector ( *An. gambiae*) mosquitoes were exposed to *B. malayi* at blood concentrations of 2,000 mf/mL or 5,000 mf/mL. E/F collection occurred at the 48- and 72-hour post-exposure time points. Irrespective of time point, qPCR analysis resulted in the detection of parasite signal from the E/F of 10 of 11 *Ae. aegypti* mosquitoes exposed at a parasitemia of 5,000 mf/mL, and from 9 of 11 *Ae. aegypti* mosquitoes exposed at a parasitemia of 2,000 mf/mL ( Figure 2A). Results for *An. gambiae* exposures were similar, with positive detection occurring in 8 of 10 samples produced from mosquitoes exposed at a parasitemia of 5,000 mf/mL and in 10 of 11 samples produced following exposure at a parasite density of 2,000 mf/mL ( Figure 2B). Consistency of detection across time points was greater when testing E/F produced by *Ae. aegypti*, occurring for six mosquitoes following exposure at 5,000 mf/mL, and six mosquitoes following exposure at 2,000 mf/mL. In E/F produced by *An. gambiae*, detection across multiple time points occurred from only three mosquitoes and one mosquito following exposures to *B. malayi* at 5,000 mf/mL and 2,000 mf/mL respectively. Unsurprisingly, for both species, mean Cq values were lower, suggesting greater concentrations of target DNA, in the E/F produced by mosquitoes exposed to higher blood concentrations of *B. malayi* ( Figure 2C). Of note, a single negative control mosquito, not exposed to *B. malayi*, did give a positive signal. Contamination, resulting in amplification, likely occurred either during mosquito rearing or during DNA extraction. The use of no-template negative controls during PCR suggests that the contamination was unlikely to have occurred during the PCR.

**Figure 3.**
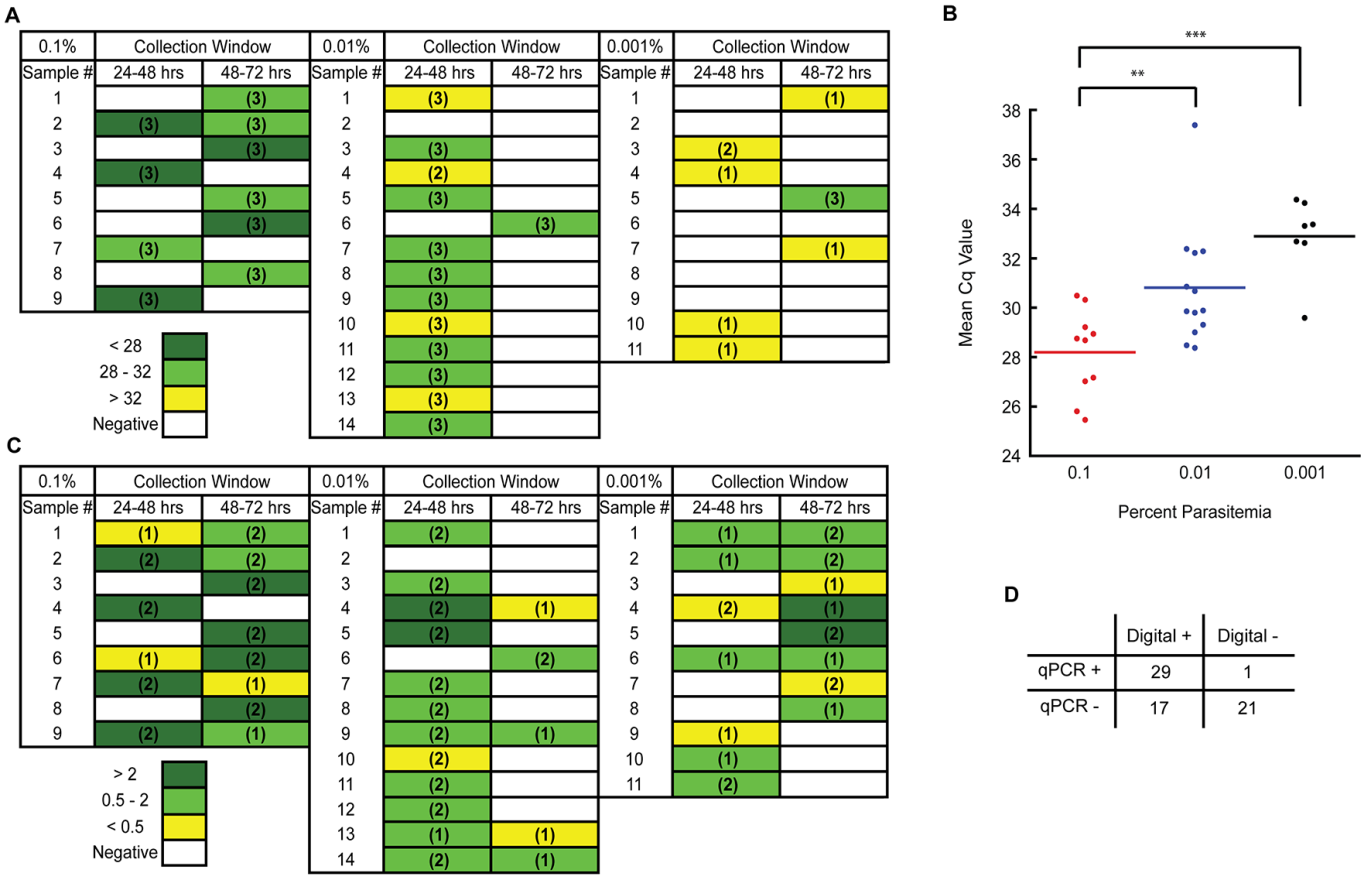
Detection windows for parasites in the excreta/feces (E/F) of mosquitoes following a *Plasmodium falciparum-*containing bloodmeal. ( **A**) Individual *Anopheles gambiae* mosquitoes were exposed to a *P. falciparum*-containing bloodmeal at a parasitemia of 5000 trophozoites/mL (0.1% parasitemia), 500 trophozoites/mL (0.01% parasitemia) or 50 trophozoites/mL (0.001% parasitemia). E/F was then collected from each mosquito, individually, in 24 hour time blocks. Collected E/F samples were then tested in triplicate qPCR reactions. Colors represent Cq values, and numbers in parenthesis indicate the number of positive qPCR replicates. ( **B**) A comparison of the mean Cq values from all qPCR-positive E/F samples (irrespective of time point) produced by mosquitoes at each tested parasitemia. Significance, as determined by the results of unpaired *t* tests, is provided. ** *p* < 0.01, *** *p* < 0.001. ( **C**) Testing, in duplicate dPCR reactions, of the same samples collected in panel **A**. Colors represent the number of positive sample wells per reaction, and numbers in parenthesis indicate the number of positive dPCR replicates. ( **D**) A comparison of E/F sample positivity as determined by qPCR and dPCR results from panels **A** and **C**.

***P. falciparum***. Following exposure of individual *An. gambiae* mosquitoes to *P. falciparum* at parasitemias of 0.1% (5000/μL), 0.01% (500/μL), and 0.001% (50/μL), E/F was collected at the 48-hour and 72-hour time points. As measured by qPCR, all nine mosquitoes exposed at 0.1% produced E/F that gave positive results: four samples were positive at the 48-hour time point, while six were positive at the 72-hour time point. Following exposure at 0.01% parasitemia, 13 of 14 mosquitoes produced E/F that gave positive qPCR results: 12 were positive at the 48-hour time point, while only one sample was positive at the 72-hour time point. Exposures at 0.001% parasitemia resulted in positive detection from the E/F produced by seven of 11 mosquitoes: four were positive at the 48-hour point, while three were positive at the 72-hour point ( Figure 3A). Interestingly, regardless of concentration, only one mosquito produced sample that was detectable at both collection time points (0.1%, sample 2). This result was in sharp contrast with findings for *B. malayi* ( Figure 2A, B). Taken together, these results may mean that deposition of parasite material occurs largely as the result of a solitary excretion event. When signal detection occurs across time points, it may be that this excretion event spans collection intervals, resulting in multiple positive time points from an isolated excretion occurrence. Whether the duration of this excretion event is longer following a *B. malayi* exposure, or these findings are chance results, remains an open question.

As expected, and as seen following *B. malayi* exposures, Cq values increased with declining numbers of parasites, suggesting greater amounts of template in the E/F produced by mosquitoes exposed to higher concentrations of pathogen ( Figure 3B). Digital PCR analysis of samples resulted in the improved overall sensitivity of detection, as more positive results were seen at the 48–72-hours time point (61.8%) compared to the qPCR results (29.4%) ( Figure 3A, C). In total, across all parasite concentrations, 17 qPCR negative samples demonstrated positivity when tested by dPCR, while only 1 sample which was qPCR positive produced a negative result by dPCR ( Figure 3D).

**Figure 4.**
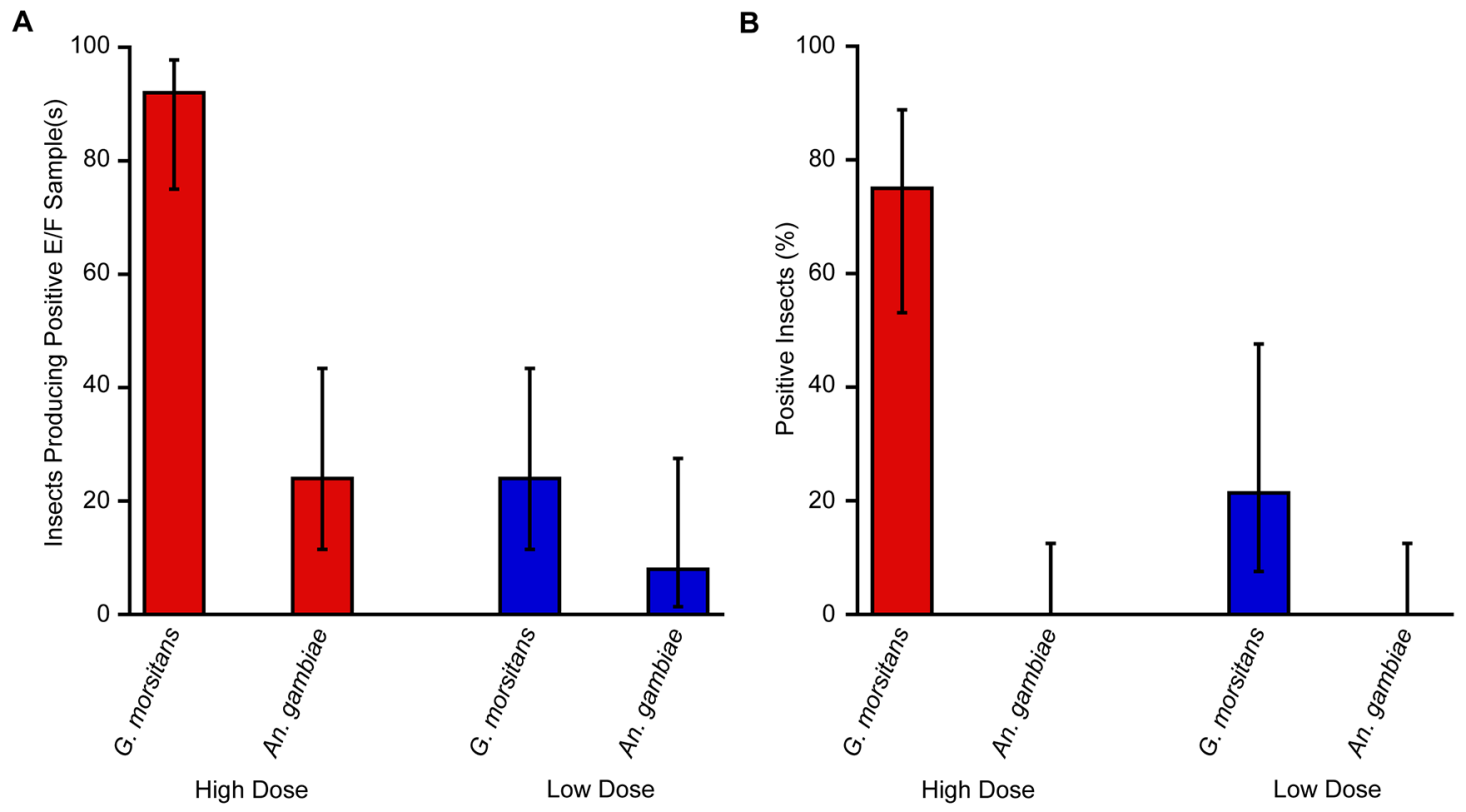
Levels of qPCR positivity in excreta/feces (E/F) samples and exposed insects following a *Trypanosoma brucei brucei*-containing bloodmeal. ( **A**) Twenty-five *G. morsitans* and 25 *An. gambiae* were provided with a *T. b. brucei*-positive bloodmeal at either high (10 ^5^ trypanosomes/mL; red) or low (10 ^3^ trypanosomes/mL; blue) parasitemias. E/F was collected from individual insects and tested by qPCR for *T. b. brucei*. Results are shown as percentages of insects producing at least one positive E/F sample from across all time points ± 95% CI. ( **B**) Following high- or low-dose exposures to *T. b. brucei* and subsequent collection and testing of E/F (depicted in panel A), DNA was extracted from *G. morsitans* midguts and *An. gambiae* carcasses. Extracts were tested for pathogen presence by qPCR and results are shown as percentages of exposed insects testing positive by qPCR ± 95% CI.

***T. b. brucei***. Following exposure to *T. b. brucei,* E/F was collected from individually housed *G. morsitans* and *A. gambiae* at 48-hour intervals. Overall, following exposure to “high dose” *T. b. brucei* (10 ^5^ trypanosomes/mL), 23 of 25 individually housed *G. morsitans* produced at least one E/F sample that was qPCR positive for parasite (92.0%). In contrast, only six of the 25 individual *An. gambiae* mosquitoes exposed to the "high dose" produced E/F which was qPCR positive for *T. b. brucei* (24.0%). Following “low dose” exposures (10 ^3^ trypanosomes/mL), six out of 25 tsetse flies produced at least one parasite-positive E/F sample (24.0%), while two mosquitoes out of the 25 exposed produced a sample that was *T. b. brucei* positive by qPCR (8.0%) ( Figure 4A). Interestingly, *T. b. brucei* detection from E/F produced by *G. morsitans* readily occurred at the 192-hour time point following both “high dose” (70.8%) and “low dose” (20.8%) exposures to parasite ( Table 1). In contrast, only the E/F produced by a single mosquito resulted in positive *T. b. brucei* detection by qPCR at a time point later than 96 hours post-infection (4.2%), and this sample was derived from an individual of “low dose” exposure ( Table 1).

**Table 1.** Percentage of qPCR-positive excreta/feces (E/F) samples collected from *Glossina morsitans* and *Anopheles gambiae* following exposure to both “high dose” and “low dose” concentrations of Trypanosoma brucei brucei. Twenty-five flies and 25 mosquitoes were individually exposed to each concentration of parasite.

	High Dose (10 ^5^ trypanosomes/mL)		Low Dose (10 ^3^ trypanosomes/mL)	
Post-Exposure Time Point (Hrs)	*G. morsitans* % Positive (95% CI)	*An. gambiae* % Positive (95% CI)	*G. morsitans* % Positive (95% CI)	*An. gambiae* % Positive (95% CI)
**48**	56.0 (35.3 – 75.0)	24.0 (11.5 – 43.4)	0.0 (0.0 – 13.3)	0.0 (0.0 – 13.3)
**96**	50.0 (31.4 – 68.6)	0.0 (0.0 – 13.3)	0.0 (0.0 – 13.3)	4.0 (0.7 – 19.5)
**144**	45.8 (27.9 – 64.9)	0.0 (0.0 – 13.8)	8.7 (2.4 – 26.8)	4.2 (0.7 – 20.3)
**192**	70.8 (50.8 – 85.1)	0.0 (0.0 – 13.8)	20.8 (9.2 – 40.5)	0.0 (0.0 – 13.8)

 Following sacrifice at the 196-hour time point, qPCR analysis of DNA extracted from *G. morsitans* midguts and *A. gambiae* carcasses was performed. Testing revealed *T. b. brucei* positivity in 15 of 20 midgut-derived samples from *G. morsitans* subjected to “high dose” exposures (75.0%), and in 3 of 14 samples collected from “low dose” individuals (21.4%). Neither “high” nor “low dose” mosquitoes produced a single *T. b. brucei*-positive carcass ( Figure 4B).

### Demonstration of high throughput detection of *P. falciparum* signal from pooled E/F

**Figure 5.**
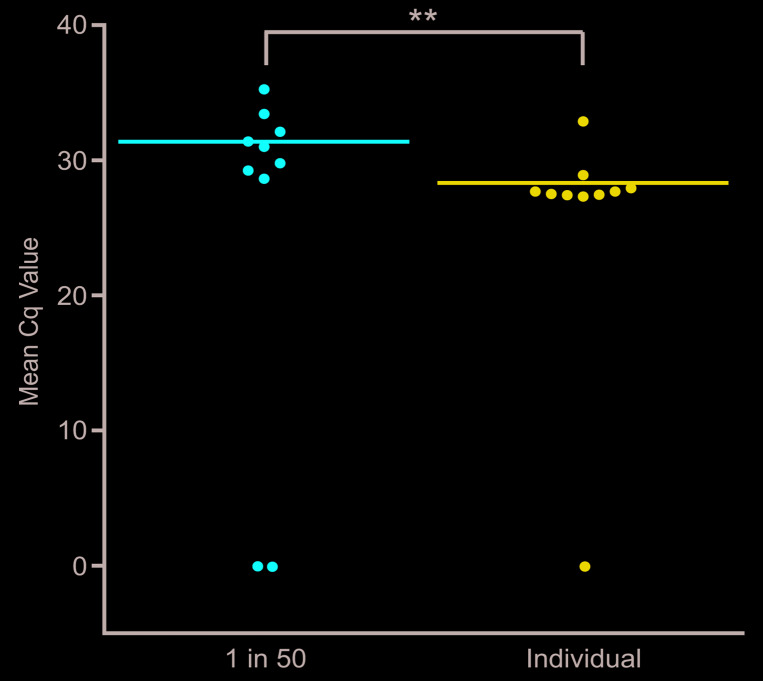
High throughput detection of *Plasmodium falciparum* signal from pooled excreta/feces (E/F). Individual *Anopheles gambiae* mosquitoes were exposed to *P. falciparum* trophozoites at a parasitemia of 0.1%. E/F was then collected from mosquitoes, either individually, or following pooling with the E/F from 49 additional *An. gambiae* mosquitoes having been exposed to a parasite-naïve bloodmeal. All E/F samples were then tested by qPCR, in triplicate reactions, for the presence of *P. falciparum* signal. Mean results from the testing of each pool are indicated. Significance, as determined by the results of unpaired *t* tests, is provided. ** *p* < 0.01.

To investigate the capacity for high throughput sampling when testing mosquito E/F for the presence of *P. falciparum* by qPCR, comparative analysis of samples containing the pooled E/F from 50 mosquitoes (49 unexposed and 1 *P. falciparum*-exposed) and mosquitoes individually exposed to *P. falciparum* was performed. Eight of 10 samples containing pooled E/F gave positive qPCR results with a mean Cq value of 31.37 for all positive samples. By comparison, nine of 10 control samples containing the E/F from individually exposed mosquitoes resulted in the detection of *P. falciparum* signal, with a mean Cq value of 28.33 for all positive samples, a difference in means that was statistically significant ( *p*=0.0067) ( Figure 5).

### Effects on parasite detection of a post-exposure second blood feeding with pathogen-naïve blood

Following an initial exposure to an infected bloodmeal, detection of *P. falciparum* signal in the E/F from all three experimental pools of 10 mosquitoes occurred. As expected, at the 144-hour post-exposure time point, signal detection was no longer possible. Following the provision of a second, uninfected bloodmeal, signal remained undetectable, indicating that such an exposure was not capable of extending, or re-initiating the post-parasite exposure collection window ( Table 2).

**Table 2.** Mean Cq values for *P. falciparum* detection in the excreta/feces (E/F) of pools of 10 exposed mosquitoes provided with a second, pathogen-naïve bloodmeal.

Sample	72 hr post 1° Exposure	144 hr post 1° Exposure	72 hr post 2° Exposure
Negative Control Pool	Undetected	Undetected	Undetected
Experimental Pool #1	33.51	Undetected	Undetected
Experimental Pool #2	31.02	Undetected	Undetected
Experimental Pool #3	36.09	Undetected	Undetected

## Discussion

Proof-of-concept work has previously demonstrated the capacity for mosquito E/F to serve as a novel, high throughput testing medium for various parasitic and viral pathogens ^44, 45, 54– 58^. However, the future utility of E/F testing will depend upon an ability to effectively and efficiently collect and test E/F from the appropriate mosquito source populations. Having previously described a novel methodology facilitating the high throughput collection of mosquito E/F ^45^, the work described here aimed to identify the characteristics of such mosquito populations through the definition of amenable mosquito species, and the determination of optimal pathogen densities, and sample pool sizes.

 For the detection of *B. malayi*, the testing of E/F from both competent and incompetent vectors consistently allowed for pathogen detection. As *An. gambiae* mosquitoes do not support *B. malayi* development, it logically follows that the ingested pathogens should undergo rapid expulsion from the mosquito in the E/F of these non-vector hosts. In contrast, as *Ae. aegypti* mosquitoes allow for *B. malayi* development, we initially hypothesized that pathogen detection in competent vector E/F may be more difficult. However, even following the exposure of an efficient vector species to mf, the percentage of ingested worms that mature to the L3 stage remains relatively low ^59^, and vector competence may not translate to efficient vector capacity under all conditions. Therefore, while possible differences in expulsion rates for parasite-derived material from competent and incompetent vector species could be anticipated, these disparities would likely be modest. Accordingly, when individual competent and incompetent vector mosquitoes were provided with a *B. malayi*-containing bloodmeal during the determination of detection limits, E/F produced by both *Ae. aegypti* and *An. gambiae* mosquitoes gave positive qPCR results from a high percentage of parasite-exposed mosquitoes, demonstrating the amenability of both populations to E/F-based collection and testing.

To an even greater extent than occurs during filarial infections of mosquitoes, the majority of parasites obtained by mosquitoes during a *P. falciparum*-containing bloodmeal are trophozoites, a lifecycle stage that is incapable of developing in the mosquito host ^60, 61^. Since trophozoites reach a developmental dead-end following a mosquito bloodmeal, it can be anticipated that they are rapidly expelled in the E/F. Likely for this reason, detection of *P. falciparum* appeared strong and consistent at both the 5,000 and 500 trophozoite/μL concentrations. It should be noted that experimental exposures to *P. falciparum* were performed using exclusively trophozoites, effectively rendering bloodmeals non-infective. For this reason, exposures were only performed using *An. gambiae* mosquitoes. Given their limited numbers within the overall *P. falciparum* population, absence of gametocytes would be unlikely to dramatically change expulsion rates of parasite-derived material. However, additional experiments in conjunction with future field-based testing will be conducted to conclusively evaluate this supposition.

Given the absence of developmental capacity of *T. b. brucei* within a mosquito, the limited detection of trypanosome signal in the E/F of exposed mosquitoes was unexpected. Since *T. b. brucei* is not believed capable of developing within the mosquito, expulsion would presumably be complete and rapid. Nonetheless, detection of *T. b. brucei* showed significantly greater promise when testing the E/F from tsetse flies, the pathogen’s vector, than when testing E/F shed by mosquitoes. Furthermore, the window for consistent *T. b. brucei* detection from tsetse fly E/F extended well past the 72-hour time point typically observed for pathogen detection from mosquitoes. This increased window could reflect the additional time required for the complete digestion of a tsetse fly bloodmeal relative to a mosquito bloodmeal ^62^, a difference that may become more discrepant in laboratory-reared flies ^63^. However, improved detection in tsetse fly E/F could also have simply been a result of increased bloodmeal volume (approximately 20 μL of blood per tsetse fly feeding ^64^ vs. 2–4 μL per *Anopheles* bloodmeal ^65^). Future work will aim to evaluate other “cross-vector” pathogen detection capacities to determine whether the observed detection challenges are unique to the *T. b. brucei-*mosquito pairing *,* or whether they are an inherent property of the “cross-vector” screening approach.

Previous work has demonstrated that the testing of mosquito E/F for the detection of *B. malayi* allows for a higher throughput of screening when compared with standard mosquito-based approaches to molecular xenomonitoring ^44^. As expected, experimental detection of *P. falciparum* demonstrated high throughput capability as well. While a direct comparison of E/F samples collected from pooled and un-pooled mosquitoes did result in a significant difference, these differences were marginal and the consistency of detection was similar ( Figure 5). Additional testing, with larger replicate numbers and increased mosquito pool sizes should help to further elucidate the true extent of this increased capacity for high throughput screening.

Previous testing, conducted by our group, of the E/F produced by laboratory-reared competent and incompetent mosquito species exposed to *B. malayi*, *P. falciparum*, or *T. b. brucei* has strongly suggested that the principal collection window for the detection of DNA from all tested pathogens occurs within the first 72 hours post-blood exposure ^45^. Results of re-feeding experiments, during which a second, parasite-naïve bloodmeal was provided to mosquitoes following an initial exposure, did not allow for an expansion of this window ( Table 2). Taken together, these results strongly suggest that when testing for the presence of parasite-derived DNA signal, E/F produced by blood-fed, resting mosquitoes represents a preferable sample population. Recent work has suggested that collection window constraints may be less important when utilizing E/F for the monitoring of viral pathogens ^54, 55^. This broadening of the collection window likely results from the ability of many viruses to replicate within the mosquito host. However, recently published data also suggests that *P. falciparum*-derived RNA is detectable in E/F, with consistency, between 15 and 19 days post pathogen exposure to Anopheles stephensi mosquitoes ^58^. Whether this finding is unique to the *An. stephensi*- *P. falciparum* relationship remains to be seen. Nonetheless, these results indicate that with the implementation of proper collection and trapping strategies, the surveillance of E/F for eukaryotic pathogens is a viable option with multiple opportunities to detect pathogen.

Despite the improved throughput of testing enabled by E/F, diagnostic sensitivity remains critical for drawing accurate conclusions from population surveys, and all possible means of maximizing sensitivity of detection should be evaluated. Accordingly, we assessed the use of dPCR as a possible methodology for improving the sensitivity of detection and for expanding temporal detection windows by comparing dPCR to standard qPCR in the evaluation of E/F samples produced by *P. falciparum*-exposed mosquitoes. While dPCR did expand the capacity for pathogen detection at reduced parasitemias, testing using the QuantStudio 3D dPCR platform is time-intensive and more costly than qPCR analysis. As such, analysis with the QuantStudio 3D dPCR platform is likely not a practical option in most E/F testing environments. However, exploration of other digital PCR platforms, and/or technological improvements may facilitate its future use, and further exploration is warranted. 

 While unlikely to replace the need for human sampling, or to completely eliminate the utility of more traditional MX approaches, E/F testing has the potential to serve as a complementary tool, filling gaps and expanding the surveillance capabilities of monitoring efforts. In addition to its possible utility as an early warning “first alert” system for detecting recrudescence or residual pathogen in post-intervention settings, the utility of E/F testing could be expanded to fill other operational gaps. In the context of LF, the rapid clearance/sterilization of adult female worms occurring under IDA is leading to questions regarding the suitability of traditional TAS surveys ^20^, as rapid pathogen clearance results in many individuals who are parasite negative but antigen positive. MX has been suggested as a possible solution to such shortcomings, as pathogen presence in the mosquito population would provide real-time evidence of recrudescence or remaining infection “hotspots”. In conjunction with such efforts, the high throughput nature of E/F testing could facilitate its usefulness as a pre-screening tool, channeling the allocation of resources for traditional MX to populations of mosquitoes demonstrating E/F positivity. One could also envision E/F testing as a mechanism facilitating *Culex* spp. monitoring for LF in urban settings, where focal transmission can occur despite the passage of TAS criteria. The relative ease of *Culex* capture in passive traps, coupled with the high throughput nature of E/F testing, could facilitate the detection of residual infections, allowing for the rapid re-introduction of intervention and establishment of appropriately targeted human surveys.

In the context of other disease settings, should E/F testing prove useful for cross-vector monitoring, envisioning its use as a mapping tool for concomitant filarial infections may also become possible. In regions of the world at risk for severe adverse events due to the presence of multiple filarial pathogens, E/F pre-screening efforts could be employed to identify the presence of parasites such as *Loa loa*, helping officials to determine where appropriate precautions such as test-and-treat strategies would be required. Coupling the high throughput nature of E/F-based testing with the growing number of examples of E/F-derived viral surveillance possibilities ^54– 57^, the potential for integrated viral/parasite monitoring efforts also becomes easy to envision. With the capacity to facilitate resource sharing and maximization, such integrated efforts are worthy of further consideration/exploration.

 Having successfully identified the characteristics of amenable mosquito populations and appropriate temporal windows for pathogen detection, the capacity for E/F testing must now be evaluated under field conditions. Ongoing work is aiming to evaluate both collection strategies and the potential for parasite detection in an operational setting. These studies will ultimately help to identify suitable use cases for E/F surveillance, facilitating deployment in appropriate situations and maximizing the utility of this novel vector screening approach.

## Data availability

### Underlying data

Open Science Framework: Laboratory evaluation of molecular xenomonitoring using mosquito excreta/feces to amplify *Plasmodium*, *Brugia*, and *Trypanosoma* DNA. https://doi.org/10.17605/OSF.IO/EWRTJ ^53^

This project contains the following underlying data:

- Compiled results_Bm_LOD_realtime.xlsx. (Raw qPCR data underlying *B. malayi* LOD experiments)
- Compiled results_Pf_LOD_realtime.xlsx. (Raw qPCR data underlying *P. falciparum* LOD experiments)
- Compiled results_Pf_re-feed_realtime.xlsx. (Raw qPCR data underlying *P. falciparum* re-feed experiments)
- Compiled results_Tbb_realtime.xlsx. (Raw qPCR data underlying experiments involving *T. b. brucei*)
- Raw data_Pf_LOD_Digital.xlsx. (Raw dPCR data underlying *P. falciparum* LOD experiments)

Data are available under the terms of the **Creative Commons Zero "No rights reserved" data waiver** (CC0 1.0 Public domain dedication).

## References

[1] Gyapong GO Owusu IO da-Costa Vroom FB: Elimination of lymphatic filariasis: current perspectives on mass drug administration. *Res Rep Trop Med.* 2018;9:25–33. 10.2147/RRTM.S125204 30050352 PMC6047620

[2] World Health Organization: Global programme to eliminate lymphatic filariasis: progress report. *Wkly Epidemiol Rec.* 2017;92:594–607. Reference Source 28984121

[3] Rabinovich RN Drakeley C Djimde AA: malERA: An updated research agenda for malaria elimination and eradication. *PLoS Med.* 2017;14(11):e1002450. 10.1371/journal.pmed.1002456 29190300 PMC5708604

[4] Franco JR Cecchi G Priotto G: Monitoring the elimination of human African trypanosomiasis: Update to 2014. *PLoS Negl Trop Dis.* 2017;11(5):e0005585. 10.1371/journal.pntd.0005585 28531222 PMC5456402

[5] World Health Organization: Progress report on the elimination of human onchocerciasis, 2015-2016. *Wkly Epidemiol Rec.* 2016;91(43):505–514. 27801998

[6] Molyneux DH: Advancing toward the Elimination of Lymphatic Filariasis. *N Engl J Med.* 2018;379(19):1871–1872. 10.1056/NEJMe1811455 30403953

[7] Ásbjörnsdóttir KH Means AR Werkman M: Prospects for elimination of soil-transmitted helminths. *Curr Opin Infect Dis.* 2017;30(5):482–488. 10.1097/QCO.0000000000000395 28700363 PMC7680933

[8] Anderson RM Turner HC Truscott JE: Should the Goal for the Treatment of Soil Transmitted Helminth (STH) Infections Be Changed from Morbidity Control in Children to Community-Wide Transmission Elimination? *PLoS Negl Trop Dis.* 2015;9(8):e0003897. 10.1371/journal.pntd.0003897 26291538 PMC4546270

[9] Tanner M Greenwood B Whitty CJ: Malaria eradication and elimination: views on how to translate a vision into reality. *BMC Med.* 2015;13:167. 10.1186/s12916-015-0384-6 26208740 PMC4514994

[10] Lyttleton C: Deviance and resistance: Malaria elimination in the greater Mekong subregion. *Soc Sci Med.* 2016;150:144–152. 10.1016/j.socscimed.2015.12.033 26751710

[11] Cohen JP Silva L Cohen A: Progress Report on Neglected Tropical Disease Drug Donation Programs. *Clin Ther.* 2016;38(5):1193–1204. 10.1016/j.clinthera.2016.02.031 27041410

[12] Thomsen EK Sanuku N Baea M: Efficacy, Safety, and Pharmacokinetics of Coadministered Diethylcarbamazine, Albendazole, and Ivermectin for Treatment of Bancroftian Filariasis. *Clin Infect Dis.* 2016;62(3):334–341. 10.1093/cid/civ882 26486704

[13] Fischer PU King CL Jacobson JA: Potential Value of Triple Drug Therapy with Ivermectin, Diethylcarbamazine, and Albendazole (IDA) to Accelerate Elimination of Lymphatic Filariasis and Onchocerciasis in Africa. *PLoS Negl Trop Dis.* 2017;11(1):e0005163. 10.1371/journal.pntd.0005163 28056015 PMC5215784

[14] King CL Suamani J Sanuku N: A Trial of a Triple-Drug Treatment for Lymphatic Filariasis. *N Engl J Med.* 2018;379(19):1801–1810. 10.1056/NEJMoa1706854 30403937 PMC6194477

[15] Weil GJ Bogus J Christian M: The safety of double- and triple-drug community mass drug administration for lymphatic filariasis: A multicenter, open-label, cluster-randomized study. *PLoS Med.* 2019;16(6):e1002839. 10.1371/journal.pmed.1002839 31233507 PMC6590784

[16] Bjerum CM Ouattara AF Aboulaye M: Efficacy and safety of a single dose of ivermectin, diethylcarbamazine and albendazole for treatment of lymphatic filariasis in Côte d'Ivoire: an open-label, randomized, controlled trial. *Clin Infect Dis.* 2019;pii: ciz1050. 10.1093/cid/ciz1050 31641754 PMC7583415

[17] Edi C Bjerum CM Ouattara AF: Pharmacokinetics, safety, and efficacy of a single co-administered dose of diethylcarbamazine, albendazole and ivermectin in adults with and without *Wuchereria bancrofti* infection in Côte d'Ivoire. *PLoS Negl Trop Dis.* 2019;13(5):e0007325. 10.1371/journal.pntd.0007325 31107869 PMC6550417

[18] Chu BK Deming M Biritwum NK: Transmission assessment surveys (TAS) to define endpoints for lymphatic filariasis mass drug administration: a multicenter evaluation. *PLoS Negl Trop Dis.* 2013;7(12):e2584. 10.1371/journal.pntd.0002584 24340120 PMC3855047

[19] Brady MA Stelmach R Davide-Smith M: Costs of Transmission Assessment Surveys to Provide Evidence for the Elimination of Lymphatic Filariasis. *PLoS Negl Trop Dis.* 2017;11(2):e0005097. 10.1371/journal.pntd.0005097 28146557 PMC5287447

[20] Srividya A Subramanian S Jambulingam P: Mapping and monitoring for a lymphatic filariasis elimination program: a systematic review. *Res Rep Trop Med.* 2019;10:43–90. 10.2147/RRTM.S134186 31239804 PMC6554002

[21] Farrell SH Anderson RM: Helminth lifespan interacts with non-compliance in reducing the effectiveness of anthelmintic treatment. *Parasit Vectors.* 2018;11(1):66 10.1186/s13071-018-2670-6 29382359 PMC5791166

[22] Harris JR Wiegand RE: Detecting infection hotspots: Modeling the surveillance challenge for elimination of lymphatic filariasis. *PLoS Negl Trop Dis.* 2017;11(5):e0005610. 10.1371/journal.pntd.0005610 28542274 PMC5453617

[23] Lau CL Sheridan S Ryan S: Detecting and confirming residual hotspots of lymphatic filariasis transmission in American Samoa 8 years after stopping mass drug administration. *PLoS Negl Trop Dis.* 2017;11(9):e0005914. 10.1371/journal.pntd.0005914 28922418 PMC5619835

[24] Srividya A Subramanian S Sadanandane C: Determinants of transmission hotspots and filarial infection in households after eight rounds of mass drug administration in India. *Trop Med Int Health.* 2018;23(11):1251–1258. 10.1111/tmi.13143 30152049

[25] Rao RU Samarasekera SD Nagodavithana KC: Comprehensive Assessment of a Hotspot with Persistent Bancroftian Filariasis in Coastal Sri Lanka. *Am J Trop Med Hyg.* 2018;99(3):735–742. 10.4269/ajtmh.18-0169 30014812 PMC6169179

[26] Sheel M Sheridan S Gass K: Identifying residual transmission of lymphatic filariasis after mass drug administration: Comparing school-based versus community-based surveillance - American Samoa, 2016. *PLoS Negl Trop Dis.* 2018;12(7):e0006583. 10.1371/journal.pntd.0006583 30011276 PMC6062125

[27] Shamsuzzaman AK Haq R Karim MJ: The significant scale up and success of Transmission Assessment Surveys ‘ *TAS*’ for endgame surveillance of lymphatic filariasis in Bangladesh: One step closer to the elimination goal of 2020. *PLoS Negl Trop Dis.* 2017;11(1):e0005340. 10.1371/journal.pntd.0005340 28141812 PMC5302837

[28] Rao RU Samarasekera SD Nagodavithana KC: Reassessment of areas with persistent Lymphatic Filariasis nine years after cessation of mass drug administration in Sri Lanka. *PLoS Negl Trop Dis.* 2017;11(10):e0006066. 10.1371/journal.pntd.0006066 29084213 PMC5679644

[29] Krisher LK Krisher J Ambuludi M: Successful malaria elimination in the Ecuador-Peru border region: epidemiology and lessons learned. *Malar J.* 2016;15(1):573. 10.1186/s12936-016-1630-x 27894320 PMC5126842

[30] Xu JW Li JJ Guo HP: Malaria from hyperendemicity to elimination in Hekou County on China-Vietnam border: an ecological study. *Malar J.* 2017;16(1):66. 10.1186/s12936-017-1709-z 28173802 PMC5297092

[31] Fernando D Wijeyaratne P Wickremasinghe R: Use of a public-private partnership in malaria elimination efforts in Sri Lanka; a case study. *BMC Health Serv Res.* 2018;18(1):202. 10.1186/s12913-018-3008-y 29566691 PMC5865373

[32] Björkman A Shakely D Ali ES: From high to low malaria transmission in Zanzibar-challenges and opportunities to achieve elimination. *BMC Med.* 2019;17(1):14. 10.1186/s12916-018-1243-z 30665398 PMC6341737

[33] Moonasar D Maharaj R Kunene S: Towards malaria elimination in the MOSASWA (Mozambique, South Africa and Swaziland) region. *Malar J.* 2016;15(1):419. 10.1186/s12936-016-1470-8 27538990 PMC4991067

[34] Lemoine JF Boncy J Filler S: Haiti’s Commitment to Malaria Elimination: Progress in the Face of Challenges, 2010-2016. *Am J Trop Med Hyg.* 2017;97(4_Suppl):43–48. 10.4269/ajtmh.16-0902 29064360 PMC5676634

[35] World Health Organization: A Framework for Malaria Elimination. Geneva: WHO Press;2017 Reference Source

[36] Franco JR Cecchi G Priotto G: Monitoring the elimination of human African trypanosomiasis: Update to 2016. *PLoS Negl Trop Dis.* 2018;12(12):e0006890. 10.1371/journal.pntd.0006890 30521525 PMC6283345

[37] Davis CN Rock KS Mwamba Miaka E: Village-scale persistence and elimination of *gambiense* human African trypanosomiasis. *PLoS Negl Trop Dis.* 2019;13(10):e0007838. 10.1371/journal.pntd.0007838 31658269 PMC6837580

[38] World Health Organization: Global Health Observatory data repository.2019 Reference Source

[39] Fauver JR Weger-Lucarelli J Fakoli LS 3rd: Xenosurveillance reflects traditional sampling techniques for the identification of human pathogens: A comparative study in West Africa. *PLoS Negl Trop Dis.* 2018;12(3):e0006348. 10.1371/journal.pntd.0006348 29561834 PMC5880402

[40] Girmay G Arega B Tesfaye D: Community-based tsetse fly control significantly reduces fly density and trypanosomosis prevalence in Metekel Zone, Northwest, Ethiopia. *Trop Amin Health Prod.* 2016;48(3):633–642. 10.1007/s11250-016-1010-0 26885985

[41] Mahamat MH Peka M Rayaisse JB: Adding tsetse control to medical activities contributes to decreasing transmission of sleeping sickness in the Mandoul focus (Chad). *PLoS Negl Trop Dis.* 2017;11(7):e0005792. 10.1371/journal.pntd.0005792 28750007 PMC5549763

[42] Percoma L Sow A Pagabeleguem S: Impact of an integrated control campaign on tsetse populations in Burkina Faso. *Parasit Vectors.* 2018;11(1):270. 10.1186/s13071-017-2609-3 29703229 PMC5923030

[43] Selby R Wamboga C Erphas O: Gambian human African trypanosomiasis in North West Uganda. Are we on course for the 2020 target? *PLoS Negl Trop Dis.* 2019;13(8):e0007550. 10.1371/journal.pntd.0007550 31412035 PMC6693741

[44] Pilotte N Zaky WI Abrams BP: A Novel Xenomonitoring Technique Using Mosquito Excreta/Feces for the Detection of Filarial Parasites and Malaria. *PLoS Negl Trop Dis.* 2016;10(4):e0004641. 10.1371/journal.pntd.0004641 27096156 PMC4838226

[45] Cook DAN Pilotte N Minetti C: A superhydrophobic cone to facilitate the xenomonitoring of filarial parasites, malaria, and trypanosomes using mosquito excreta/feces [version 2; peer review: 2 approved]. *Gates Open Res.* 2017;1:7. 10.12688/gatesopenres.12749.2 29377042 PMC5781187

[46] Halliday A Guimaraes AF Tyrer HE: A murine macrofilaricide pre-clinical screening model for onchocerciasis and lymphatic filariasis. *Parasit Vectors.* 2014;7:472. 10.1186/s13071-014-0472-z 25338621 PMC4212127

[47] MacGregor P Rojas F Dean S: Stable transformation of pleomorphic bloodstream form *Trypanosoma brucei*. *Mol Biochem Parasitol.* 2013;190(2):60–62. 10.1016/j.molbiopara.2013.06.007 23835071 PMC4003390

[48] Cunningham LJ Lingley JK Haines LR: Illuminating the Prevalence of *Trypanosoma brucei s.l.* in *Glossina* Using LAMP as a Tool for Xenomonitoring. *PLoS Negl Trop Dis.* 2016;10(2):e0004441. 10.1371/journal.pntd.0004441 26890882 PMC4758712

[49] Rau RU Weil GJ Fischer K: Detection of *Brugia* parasite DNA in human blood by real-time PCR. *J Clin Microbiol.* 2006;44(11):3887–3893. 10.1128/JCM.00969-06 16957038 PMC1698366

[50] Zulch MF Pilotte N Grant JR: Selection and exploitation of prevalent, tandemly repeated genomic targets for improved real-time PCR-based detection of *Wuchereria bancrofti* and *Plasmodium falciparum* in mosquitoes. *PLoS One.* 2020;15(5):e0232325. 10.1371/journal.pone.0232325 32357154 PMC7194414

[51] Newcombe RG: Two-sided confidence intervals for the single proportion: comparison of seven methods. *Stat Med.* 1998;17(8):857–872. 10.1002/(sici)1097-0258(19980430)17:8<857::aid-sim777>3.0.co;2-e 9595616

[52] Wilson EB: Probable Inference, the Law of Succession, and Statistical Inference. *J Am Stat Assoc.* 1927;22(158):209–212. 10.2307/2276774

[53] Pilotte N: Laboratory evaluation of molecular xenomonitoring using mosquito excreta/feces to amplify Plasmodium, Brugia, and Trypanosoma DNA.2019 10.17605/OSF.IO/EWRTJ PMC7308644 32596646

[54] Fontaine A Jiolle D Moltini-Conclois I: Excretion of dengue virus RNA by *Aedes aegypti* allows non-destructive monitoring of viral dissemination in individual mosquitoes. *Sci Rep.* 2016;6:24885. 10.1038/srep24885 27117953 PMC4846815

[55] Ramírez AL Hall-Mendelin S Doggett SL: Mosquito excreta: A sample type with many potential applications for the investigation of Ross River virus and West Nile virus ecology. *PLoS Negl Trop Dis.* 2018;12(8):e0006771. 10.1371/journal.pntd.0006771 30169512 PMC6136815

[56] Ramírez AL Hall-Mendelin S Hewitson GR: Stability of West Nile Virus (Flaviviridae: Flavivirus) RNA in Mosquito Excreta. *J Med Entomol.* 2019;56(4):1135–1138. 10.1093/jme/tjz044 30937448

[57] Meyer DB Ramírez AL van den Hurk AF: Development and Field Evaluation of a System to Collect Mosquito Excreta for the Detection of Arboviruses. *J Med Entomol.* 2019;56(4):1116–1121. 10.1093/jme/tjz031 30945738

[58] Ramírez AL van den Hurk AF Mackay IM: Malaria surveillance from both ends: concurrent detection of *Plasmodium falciparum* in saliva and excreta harvested from *Anopheles* mosquitoes. *Parasit Vectors.* 2019;12(1):355. 10.1186/s13071-019-3610-9 31319880 PMC6639908

[59] Stolk WA Van Oortmarssen GJ Subramanian S: Assessing density dependence in the transmission of lymphatic filariasis: uptake and development of *Wuchereria bancrofti* microfilariae in the vector mosquitoes. *Med Vet Entomol.* 2004;18(1):57–60. 10.1111/j.0269-283x.2004.0470.x 15009446

[60] Talman AM Domarle O McKenzie FE: Gametocytogenesis: the puberty of *Plasmodium falciparum*. *Malar J.* 2004;3:24. 10.1186/1475-2875-3-24 15253774 PMC497046

[61] Eichner M Diebner HH Molineaux L: Genesis, sequestration and survival of *Plasmodium falciparum* gametocytes: parameter estimates from fitting a model to malariatherapy data. *Trans R Soc Trop Med Hyg.* 2001;95(5):497–501. 10.1016/s0035-9203(01)90016-1 11706658

[62] Weiss BL Maltz MA Vigneron A: Colonization of the tsetse fly midgut with commensal *Kosakonia cowanii* Zambiae inhibits trypanosome infection establishment. *PLoS Pathog.* 2019;15(2):e1007470. 10.1371/journal.ppat.1007470 30817773 PMC6394900

[63] Jeffery GM: Blood meal volume in *Anopheles quadrimaculatus*, *A. albimanus* and *Aedes aegypti*. *Exp Parasitol.* 1956;5(4):371–375. 10.1016/0014-4894(56)90021-2 13344500

[64] Olembo NK Nguu EK Ochanda JO: Inhibition of bloodmeal digestion in *Glossina morsitans* fed on rabbits immunized with tsetse midgut homogenate. *East Afr Med J.* 1994;71(10):651–655. 7821245

[65] Langley PA: The control of digestion in the tsetse fly, *Glossina morsitans*: A comparison between field flies and flies reared in captivity. *J Insect Physiol.* 1967;13(3):477–486. 10.1016/0022-1910(67)90086-8 6081063

