# Supplementary material for: Laboratory evaluation of molecular xenomonitoring using mosquito and tsetse fly excreta/feces to amplify Plasmodium, Brugia, and Trypanosoma DNA
Source: Gates Open Res. 2020 Jun 3;3:1734. Originally published 2019 Dec 19. [Version 2] doi: 10.12688/gatesopenres.13093.2 (PMC7308644; doi:10.12688/gatesopenres.13093.2)
Supplement: Peer review history

# Peer review: Reviewer response for version 2

**DOI:** 10.21956/gatesopenres.14332.r28897

**Published:** 2020-06-17

**Authors:** Ana L. Ramirez

**Affiliations:** 1 College of Public Health, Medical and Veterinary Sciences, James Cook University, Cairns, Australia

The authors have made changes that substantially improve the manuscript.

Is the work clearly and accurately presented and does it cite the current literature?

Yes

If applicable, is the statistical analysis and its interpretation appropriate?

Yes

Are all the source data underlying the results available to ensure full reproducibility?

Yes

Is the study design appropriate and is the work technically sound?

Yes

Are the conclusions drawn adequately supported by the results?

Yes

Are sufficient details of methods and analysis provided to allow replication by others?

Partly

Reviewer Expertise:

Vector biology, virology, arbovirus surveillance.

I confirm that I have read this submission and believe that I have an appropriate level of expertise to confirm that it is of an acceptable scientific standard.

# Peer review: Reviewer response for version 1

**DOI:** 10.21956/gatesopenres.14242.r28626

**Published:** 2020-03-09

**Authors:** Hans-Peter Fuehrer

**Affiliations:** 1 Institute of Parasitology, Department of Pathobiology, University of Veterinary Medicine Vienna, Vienna, Austria

Pilotte *et al.* describe the use of excreta/faeces of mosquitoes and tsetse flies after experimental exposure to *Brugia malayi*, *Plasmodium falciparum* and *Trypanosoma brucei brucei* for molecular xeno-monitoring.

 The manuscript is of very good quality and of interest. Only few modifications are recommended:

- Title: Maybe include tsetse-flies in the title.
- Methods: Please name “strains” or “origin” of the vectors.
- Page 4: Brugia experiments: Change „the“ to „then“ – at: „Cones were then placed…“.
- Entire manuscript: Please use Ae. for Aedes and An. for Anopheles instead of A. (e.g. Figure 5).
- Discussion: It is recommended to add a sentence that “vector competence” is not a proof for true vector capacity in the field.

Is the work clearly and accurately presented and does it cite the current literature?

Yes

If applicable, is the statistical analysis and its interpretation appropriate?

Yes

Are all the source data underlying the results available to ensure full reproducibility?

Yes

Is the study design appropriate and is the work technically sound?

Yes

Are the conclusions drawn adequately supported by the results?

Yes

Are sufficient details of methods and analysis provided to allow replication by others?

Yes

Reviewer Expertise:

Mosquitoes and mosquito borne diseases; Molecular xeno-monitoring

I confirm that I have read this submission and believe that I have an appropriate level of expertise to confirm that it is of an acceptable scientific standard.

# Peer review: Reviewer response for version 1

**DOI:** 10.21956/gatesopenres.14242.r28423

**Published:** 2020-01-28

**Authors:** Ana L. Ramirez

**Affiliations:** 1 College of Public Health, Medical and Veterinary Sciences, James Cook University, Cairns, Australia

This paper by Nils Pilotte and co-authors describe the evaluation of using mosquito excreta/feces for xenomonitoring applications. The use of mosquito excreta as a tool for molecular xenomonitoring of parasitic diseases has clear applications for disease surveillance and the evaluation of intervention programmes. The authors evaluated key factors that can have an effect once the methodology is deployed to the field, such as the limits of detection, throughput of testing and the effect of subsequent bloodmeals after exposure using different vector/parasite models. Additionally, this is the first study evaluating molecular detection of *T. b. brucei* in excreta from tsetse flies. The article is well written, and the methods are generally clear. All the data generated from the study has been deposited in a data repository. However, some issues in the results need to be addressed (clarification of pooled/individual samples and checking the consistency between data presented in figures/tables and in-text).

**Introduction**

 Consider removing acronyms that are only used once (i.e. in the second paragraph of the introduction “operational research (OR)”).

**Methods**

 Most of the information in the methods section is presented in a clear and detailed manner. A few observations:

- Page 4 - Tsetse flies. “Flies were reared from larvae..” Add species at the first mention “ *Glossina morsitans* flies were reared from larvae”.
- Page 4 - Were parasite densities determined for *B. malayi* and *P. falciparum* too or just for the trypanosomes?
- Page 4 - For the collection of excreta/feces: were the GenSaver DNA cards used whole or did you cut a smaller piece?
- Page 5 - Real-time PCR: “Quantitative real-time PCR testing for the presence/absence..” --> mention what was tested by qPCR (DNA extracts from E/F, fly midguts and whole mosquitoes and flies?). Same for Digital PCR (only DNA extracts from E/F).
- Page 5 - Mention the controls used for each qPCR assay and the Cq used as a cut-off.
- Page 6 - Limits of detection for parasite signal from pooled E/F: How many mosquitoes were used to collect pooled E/F?
- Page 6 - Both hours and days are used through the manuscript (i.e “144 hours post-exposure”, then “at six days post feeding”. Consider sticking to hours or days for consistency.

**This is just a suggestion** but given that the methodology has different components it could be re-structured to make it easier to follow by first describing the insects and parasites, then moving to the exposure, then describing the design of each experiment and finally describing how the samples were tested. Something like:

- Insect rearing
  - Mosquitoes
  - Tse tse flies
- Parasites
  - *B. malayi*
  - *P. falciparum*
  - *T. b. brucei*
- Blood feeding
  - Move here the sections where you describe the exposure for all of the above (i.e “experimental exposures were performed as previously described. Briefly, adult female mosquitoes…”
- Collection of excreta/feces (keep it as it is).
- Limits of detection for parasite signal from pooled E/F.
- Demonstration of high throughput detection of P. falciparum signal from pooled E/F.
- Effects on parasite detection of a second blood feeding with pathogen-naïve blood.
- Isolation of tsetse fly midguts and preparation for DNA extraction.
- DNA extraction from E/F.
- DNA extraction from DNA from mosquitoes and tsetse flies.
- Real-time PCR.
- Digital PCR.
- Statistical analysis.

**Results**

- Page 7 - Limits of detection for parasite signal from pooled EF: Check that the numbers in text match the info in the figures/tables. “Consistency of detection across time points (…) and FIVE mosquitoes following exposure at 2,000 mf/mL”. In the table, there are SIX mosquitoes with detection at both time points. “In E/F produced by *A. gambiae* detection across multiple time points occurred from only TWO mosquitoes and one mosquito following…” in the table there are 3 mosquitoes with detection at both time points.
- Page 7 - Limits of detection for parasite signal from pooled EF: The text in this section references Figure 2. However, the caption indicates that “E/F was collected from each mosquito”. It is not clear if results from E/F from individual mosquitoes is being shown or if correspond to pooled samples. If it is pooled, indicate how many and change the figure. If it is from individual mosquitoes removed “pooled” from the section.
- Page 7 - Limits of detection for parasite signal from pooled EF: “unsurprisingly, mean Cq values were lower, suggesting greater concentration of target DNA..” --> “unsurprisingly, for both species, Cq values were lower, suggesting…”
- Page 7 - Figure 2C: consider changing the x-axis labels to horizontal and add units (Vector species and parasite density (mf/mL).
- Page 8 - *P. falciparum*: Stick to either percentage or trophozoites/mL. In the text it is shown mostly as percentage so maybe change the column headings in Figure 3A and 3C to match.
- Page 8 - “Interestingly, only one mosquito produce sample that was detectable at both collection time points” change to “Interestingly, regardless of concentration, only one mosquito produce sample that was detectable at both collection time points”
- Page 8 - The following statement is not clear “When signal detection occurs across time points, it may be that this event spans collection intervals, resulting in multiple positive time points from an isolated excretion occurrence”. Is the event the act of excreting parasite?
- Page 8 - Figure 2B shows a comparison of mean Cq values irrespective of timepoints, was a comparison done of Cq values between time points?
- Page 9 - First paragraph: consider showing the percentage of samples with positive results from qPCR vs dPCR.
- Page 9 - *T.b.brucei*: The results from E/F collected after 48 and 144 hours are not mentioned in text (they appear in Table 1).
- Table 1: check the numbers from the table, as they don’t match the results shown in text (96 h. Also add N. In the title change “trypanosomes” to “ *T. b. brucei*”.
- Page 9 - add percentages when describing the data to be consistent “In contrast, only six of the 25 individual *A. gambiae*…”--> “In contrast, only six of the 25 individual *A. gambiae *(24%)…”
- Page 9 - Figure 4. The legend says “qPCR positivity in excreta/feces POOLS and exposed insects” however in the text it is mentioned “individually housed *G. morsitans*”. Again, it is not clear if results from E/F from individual flies is being shown or if correspond to pooled samples. If it is pooled, indicate how many and change the figure. If it is from individual mosquitoes removed “pooled” from the section.
- Page 9 - Figure 4. Mention what time point is being shown (i.e “samples collected after 96 hours of exposure”). Also, consider rotating the labels in x-axis to either horizontal or 45 degrees.
- Page 9 - “Following sacrifice at the 196-hour time point” add percentages to “15 out of 20” and “3 out of 14” (75%, 21%).
- Page 10 - First paragraph: Is the difference between mean Cq values from positive samples pooled with negative ones and control samples significant?
- Page 10 - Figure 5: change “E/F” was then collected from mosquitoes, either individually, or following pooling with 49 additional *A. gambiae *mosquitoes having…” to “E/F” was then collected from mosquitoes, either individually, or following pooling with THE E/F FROM 49 additional *A. gambiae* mosquitoes having…"

**Discussion**

- Page 11 - Paragraph 3: The authors observed that for flies, the window of detection extended past 72 hours. It is known that digestion of bloodmeals by mosquitoes generally is finalized by 72 hours. How long does it take for a tsetse fly to digest its bloodmeal? If it’s longer, perhaps this would explain why the parasite is still detectable in their excreta after 72 hours.
- Page 11 - Paragraph 5: The authors state that previous studies have suggested that the collection window for detection of *P. falciparum* occurs within 72-hours post-exposure. However, in a study conducted by our group using competent *An. stephensi* mosquitoes, we have observed excretion of *P. falciparum* genetic material from day 4 to 14 post-exposure (intermittently) and continually from day 15 to 19 (Ramirez 2019) ^1^.

**General comments:**

- Check that controls are mentioned in the methodology.
- Make sure to make a clear distinction when pooled excreta and the excreta from individual insects is being used. Check figure titles, legends and in-text results. Also, when pooled excreta is being used, indicate the pool size.
- Keep it consistent with the percentages.
- Check the data in Figure 2 and Table 1.

Is the work clearly and accurately presented and does it cite the current literature?

Yes

If applicable, is the statistical analysis and its interpretation appropriate?

Yes

Are all the source data underlying the results available to ensure full reproducibility?

Yes

Is the study design appropriate and is the work technically sound?

Yes

Are the conclusions drawn adequately supported by the results?

Yes

Are sufficient details of methods and analysis provided to allow replication by others?

Partly

Reviewer Expertise:

Vector biology, virology, arbovirus surveillance.

I confirm that I have read this submission and believe that I have an appropriate level of expertise to confirm that it is of an acceptable scientific standard, however I have significant reservations, as outlined above.

## References

[1] : Malaria surveillance from both ends: concurrent detection of Plasmodium falciparum in saliva and excreta harvested from Anopheles mosquitoes. *Parasit Vectors*.2019;12(1) : 10.1186/s13071-019-3610-9 355 10.1186/s13071-019-3610-9 31319880PMC6639908
